# Supplementary material for: Development of Electromagnetic-Wave-Shielding Polyvinylidene Fluoride–Ti3C2Tx MXene–Carbon Nanotube Composites by Improving Impedance Matching and Conductivity
Source: Nanomaterials (Basel). 2023 Jan 19;13(3):417. doi: 10.3390/nano13030417 (PMC9921545; doi:10.3390/nano13030417)
Supplement: Supplementary file 1 [file nanomaterials-13-00417-s001.zip › nanomaterials-2088508-supplementary.pdf]

nanomaterials

Supplementary Materials

Development of Electromagnetic-Wave-Shielding Polyvinylidene Fluoride–$Ti_3C_2T_x$ MXene–Carbon Nanotube Composites by Improving Impedance Matching and Conductivity

Qimei Zhang [1,2], Jian Cui [1], Shuai Zhao [1], Guangfa Zhang [1], Ailin Gao [1] and Yehai Yan [1,*]

[1] Key Laboratory of Rubber-Plastics, Ministry of Education/Shandong Provincial Key Laboratory of Rubber-Plastics, School of Polymer Science and Engineering, Qingdao University of Science and Technology, Qingdao 266042, China
[2] School of Materials and Environmental Engineering, Chizhou University, Chizhou 247000, China
* Correspondence: yhyan@qust.edu.cn

1. Preparation

Preparation of PVDF/MXene/SWCNTs Composite

The powders for the PVDF/MXene5 and PVDF/SWCNTs2 composite microspheres were mixed in equal quantities and compressed. After cooling, the PVDF/MXene2.5/SWCNTs1 composite was obtained.

Preparation of PVDF-SWCNTs1-MXene2.5 Composite

Two gram PVDF@SWCNTs1 powders treated with PEI dropped into MXene (150ml, 1.67mg·ml^{-1}), stirred, filtration, washed and dried. After compression-molding, the PVDF-SWCNTs1-MXene2.5 composite is harvested. Schematic illustration of the preparation of PVDF/MXene/SWCNTs and PVDF/MXene/SWCNTs composites are shown in Figure S1c and d.

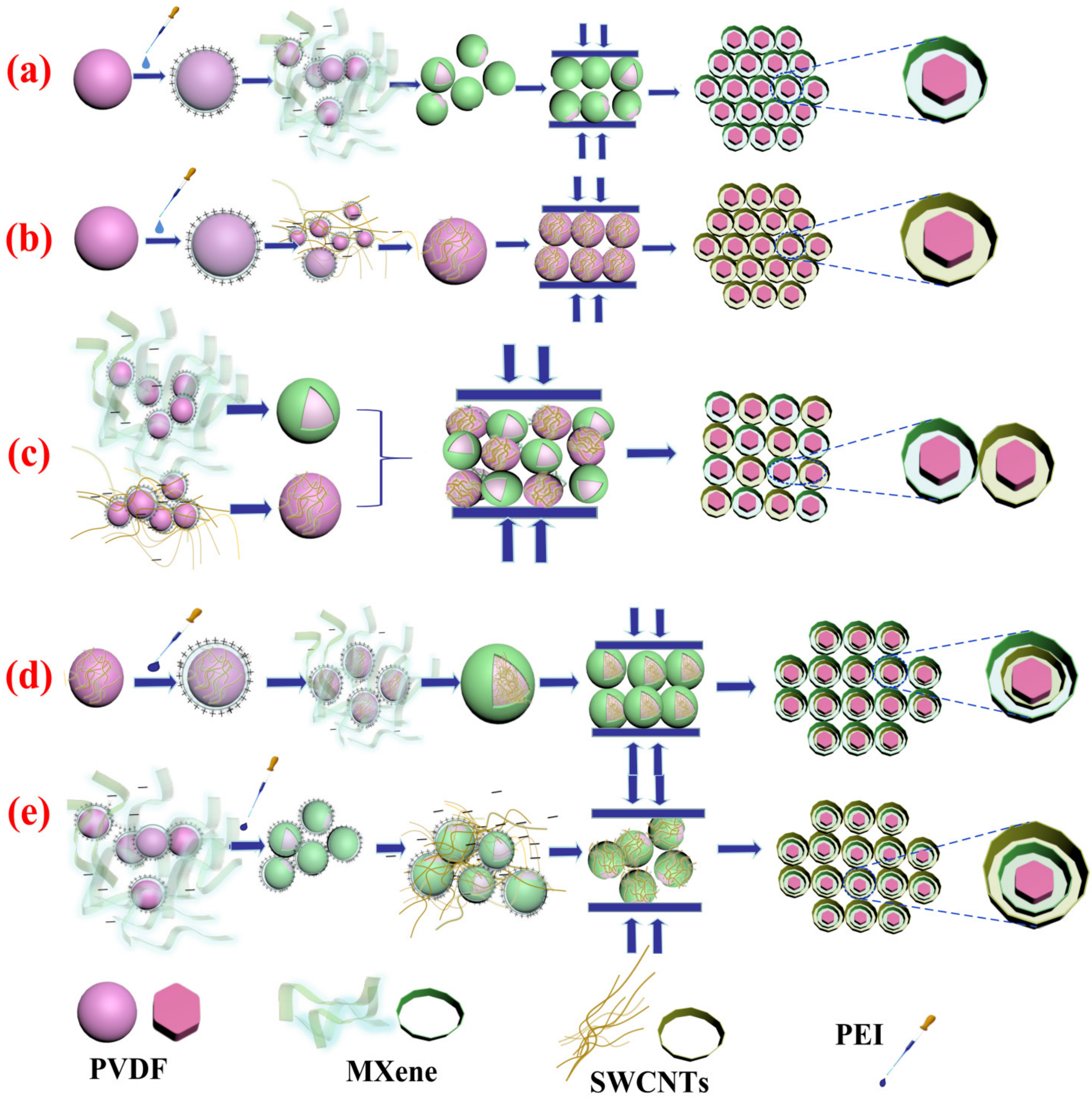

Figure S1. Schematic illustration of the preparation of segregated **(a)** PVDF/MXene, **(b)**PVDF/SWCNTs, **(c)** PVDF/MXene/SWCNTs, **(d)**PVDF-SWCNTs-MXene, and **(e)** PVDF-MXene-SWCNTs composites by electrostatic flocculation process.

Photographs of PVDF/MXene5, PVDF/SWCNTs2, PVDF-SWCNTs1-MXene2.5, and PVDF-MXene2.5-SWCNTs1 microspheres and PVDF/MXene2.5/SWCNTs1 composite are shown in Figure S2.

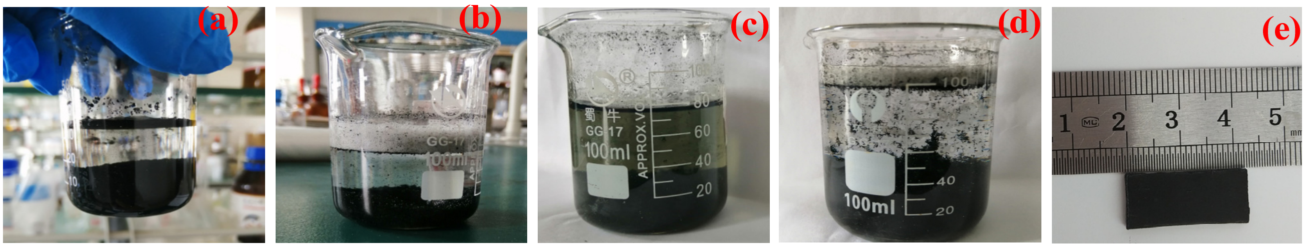

Figure S2. Photos of newly prepared **(a)** PVDF/MXene5, **(b)** PVDF/SWCNTs2, **(c)**PVDF-SWCNTs-MXene, and **(d)** PVDF-MXene-SWCNTs composite microspheres. **(e)** electromagnetic shielding sample of PVDF/MXene2.5/SWCNTs1 composite by waveguide method at 8.2–12.4 GHz.

2. Zeta Potential Results

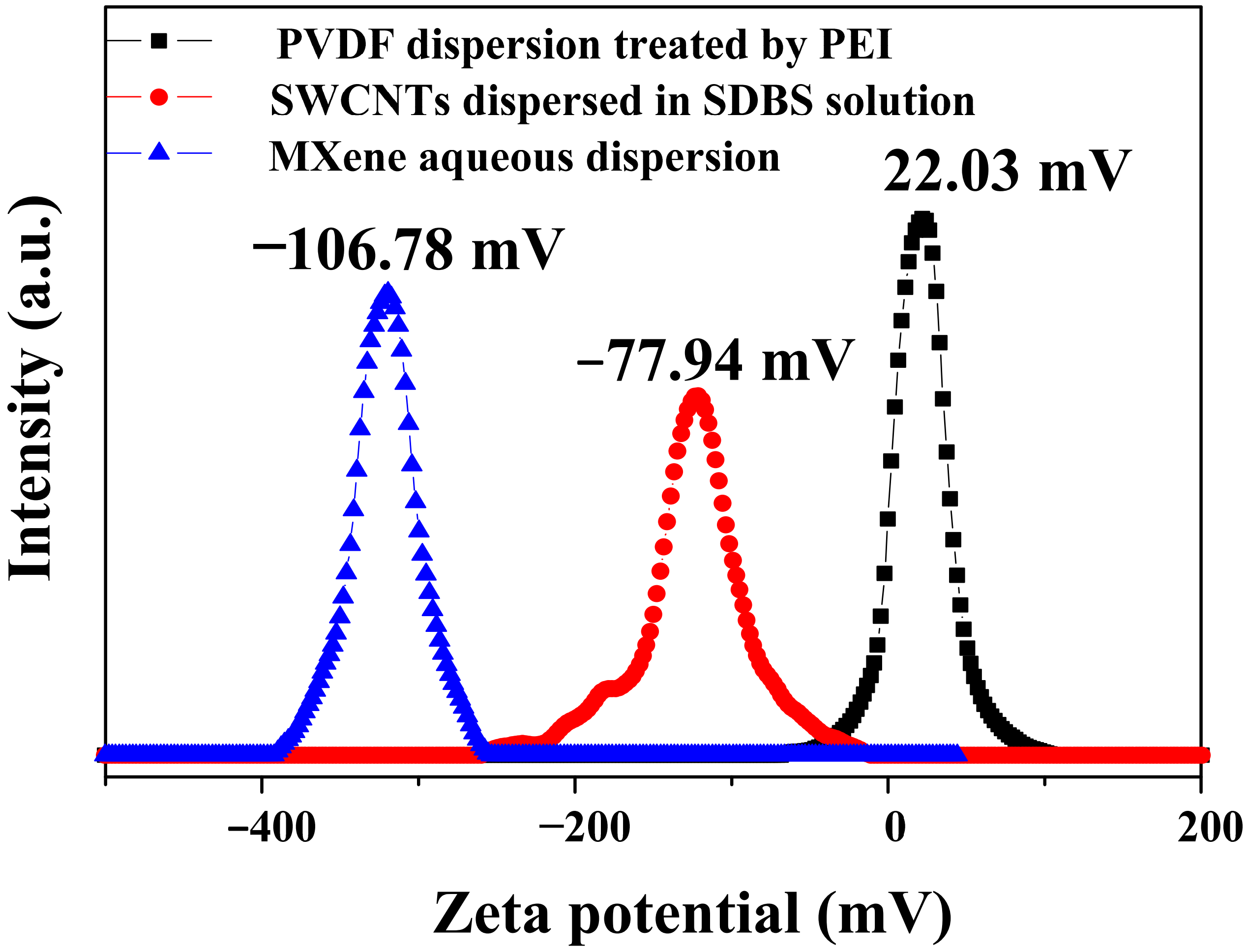

Figure S3. Zeta potential of **(a)** MXene aqueous dispersion; **(b)** PVDF microspheres aqueous dispersion treated by PEI ; and **(c)** SWCNTs dispersed by SDBS.

3. Morphologies of PVDF-SWCNTs-MXene and PVDF-MXene-SWCNTs Microspheres

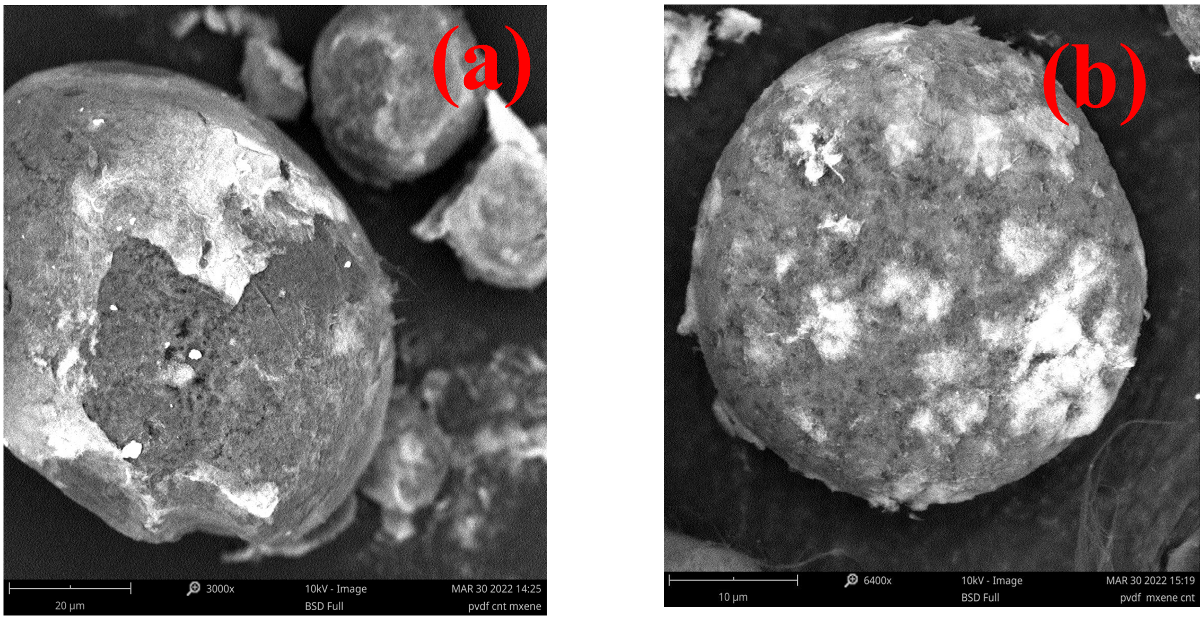

Figure S4. SEM of **(a)** PVDF-SWCNTs1-MXene2.5 and **(b)** PVDF-MXene2.5-SWCNTs1 composite microspheres.

4. The Permeability Real Part and Maginary Part of PVDF/MXene5, PVDF/SWCNTs2, PVDF/MXene2.5/SWCNTs1, PVDF-SWCNTs1-MXene2.5, and PVDF-MXene2.5-SWCNTs1.

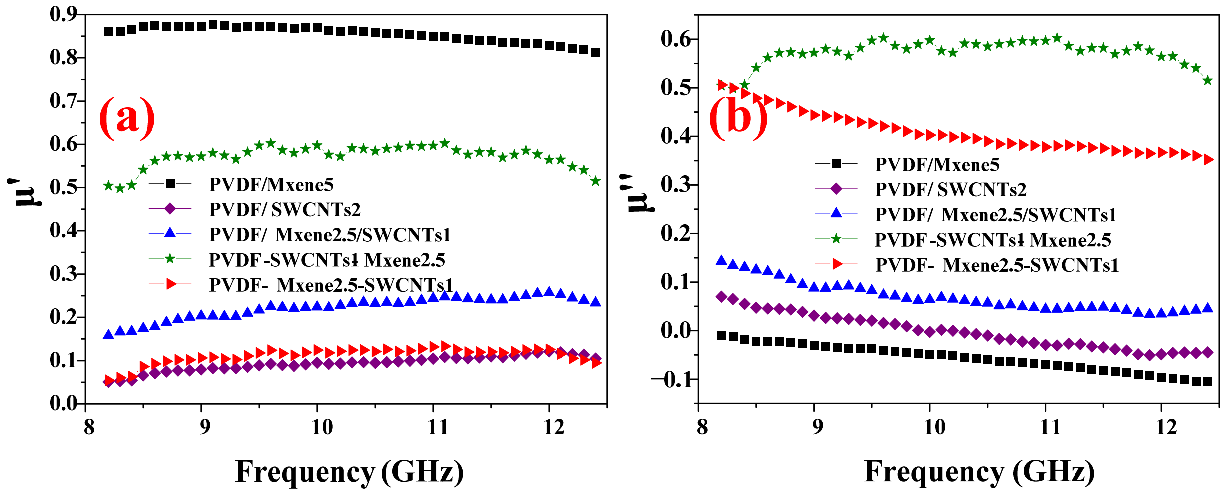

Figure S5. **(a)** Permeability real (μ') and **(b)** imaginary (μ'') parts of PVDF/MXene5, PVDF/SWCNTs2, PVDF/MXene2.5/SWCNTs1, PVDF-SWCNTs1-MXene2.5, and PVDF-MXene2.5-SWCNTs1.
